# Supplementary material for: LPCAT1-TERT fusions are uniquely recurrent in epithelioid trophoblastic tumors and positively regulate cell growth
Source: PLoS One. 2021 May 25;16(5):e0250518. doi: 10.1371/journal.pone.0250518 (PMC8148365; doi:10.1371/journal.pone.0250518)
Supplement: S1 Fig — ETT-1 demonstrated gain of chromosome 5 but also showed reduced probe intensities consistent with low-level loss corresponding to 3`LPCAT1, 5`TERT, and all intervening genes. (PPTX) [file pone.0250518.s001.pptx]

## Slide 1
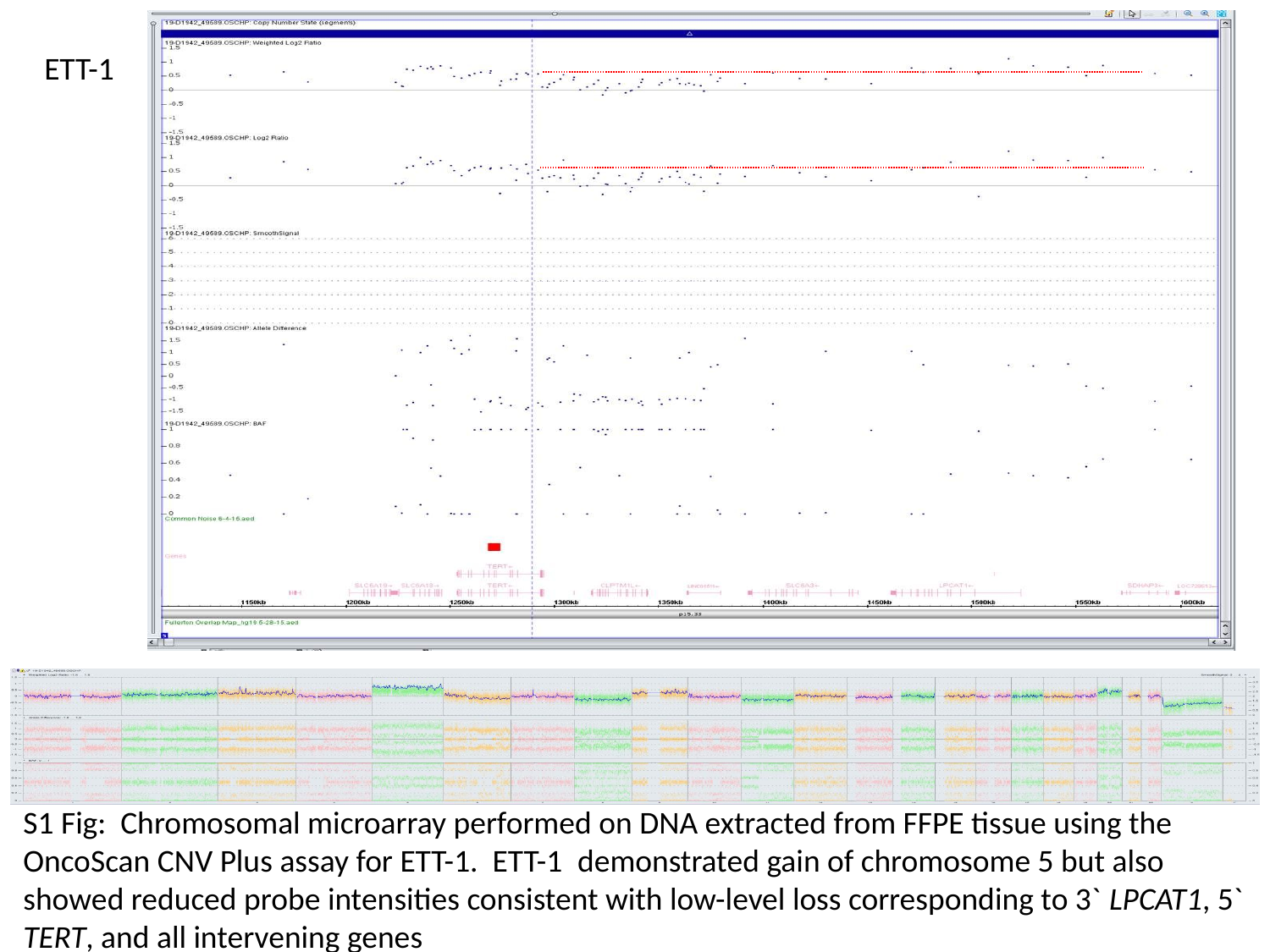

ETT-1
S1 Fig: Chromosomal microarray performed on DNA extracted from FFPE tissue using the OncoScan CNV Plus assay for ETT-1. ETT-1  demonstrated gain of chromosome 5 but also showed reduced probe intensities consistent with low-level loss corresponding to 3` LPCAT1, 5` TERT, and all intervening genes
