# Supplementary material for: LPCAT1-TERT fusions are uniquely recurrent in epithelioid trophoblastic tumors and positively regulate cell growth
Source: PLoS One. 2021 May 25;16(5):e0250518. doi: 10.1371/journal.pone.0250518 (PMC8148365; doi:10.1371/journal.pone.0250518)
Supplement: S2 Fig — ETT-2 appeared to have gain of chromosome 5 as well as reduced probe intensities supportive of a two-copy genomic deletion underlying the LPCAT1-TERT fusion. (PPTX) [file pone.0250518.s002.pptx]

## Slide 1
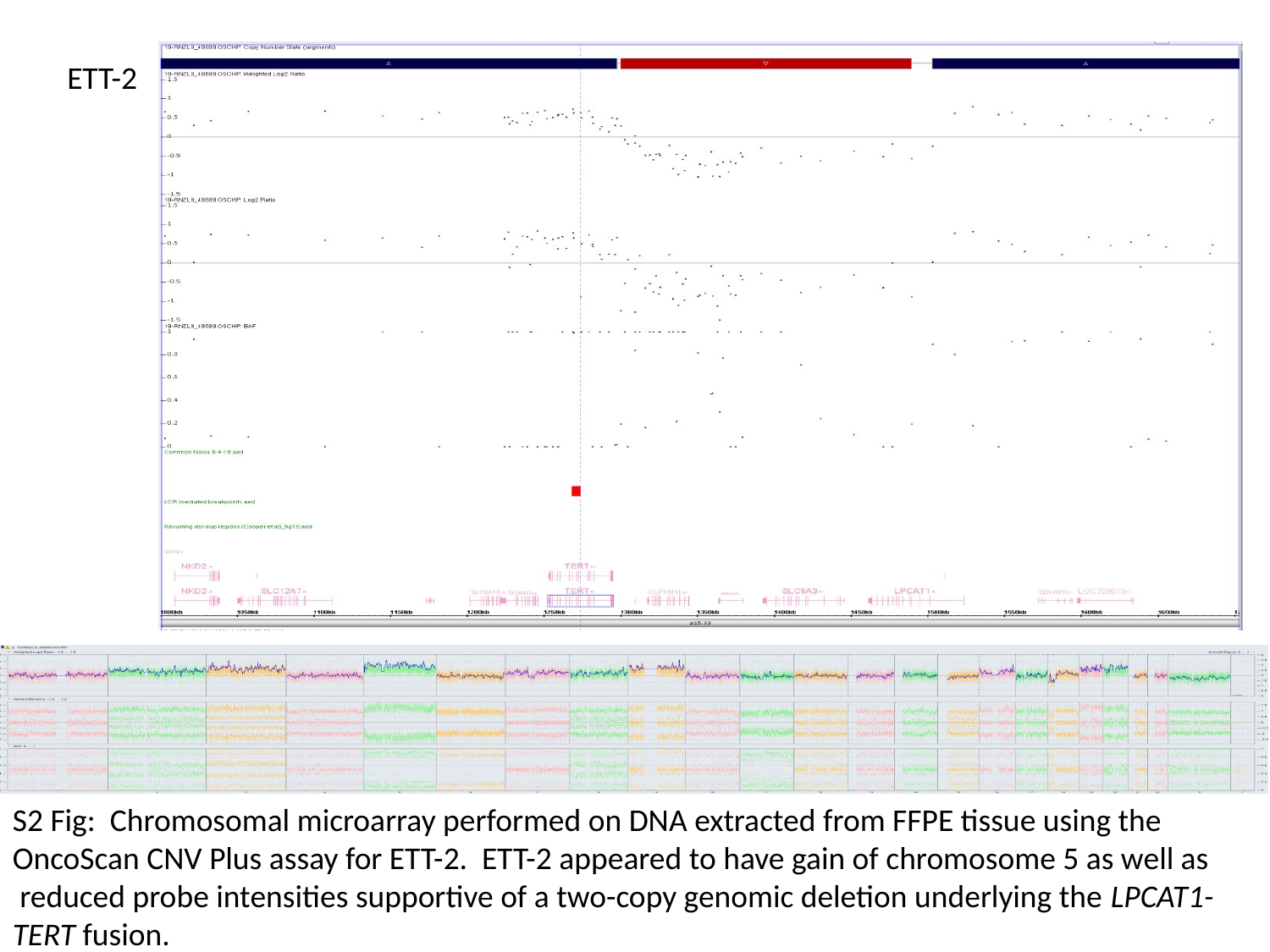

ETT-2
S2 Fig: Chromosomal microarray performed on DNA extracted from FFPE tissue using the OncoScan CNV Plus assay for ETT-2. ETT-2 appeared to have gain of chromosome 5 as well as  reduced probe intensities supportive of a two-copy genomic deletion underlying the LPCAT1-TERT fusion.
