# Supplementary material for: LPCAT1-TERT fusions are uniquely recurrent in epithelioid trophoblastic tumors and positively regulate cell growth
Source: PLoS One. 2021 May 25;16(5):e0250518. doi: 10.1371/journal.pone.0250518 (PMC8148365; doi:10.1371/journal.pone.0250518)
Supplement: S6 Fig — PSN-3 could not be assessed due to sample quality issues. (PPTX) [file pone.0250518.s006.pptx]

## Slide 1
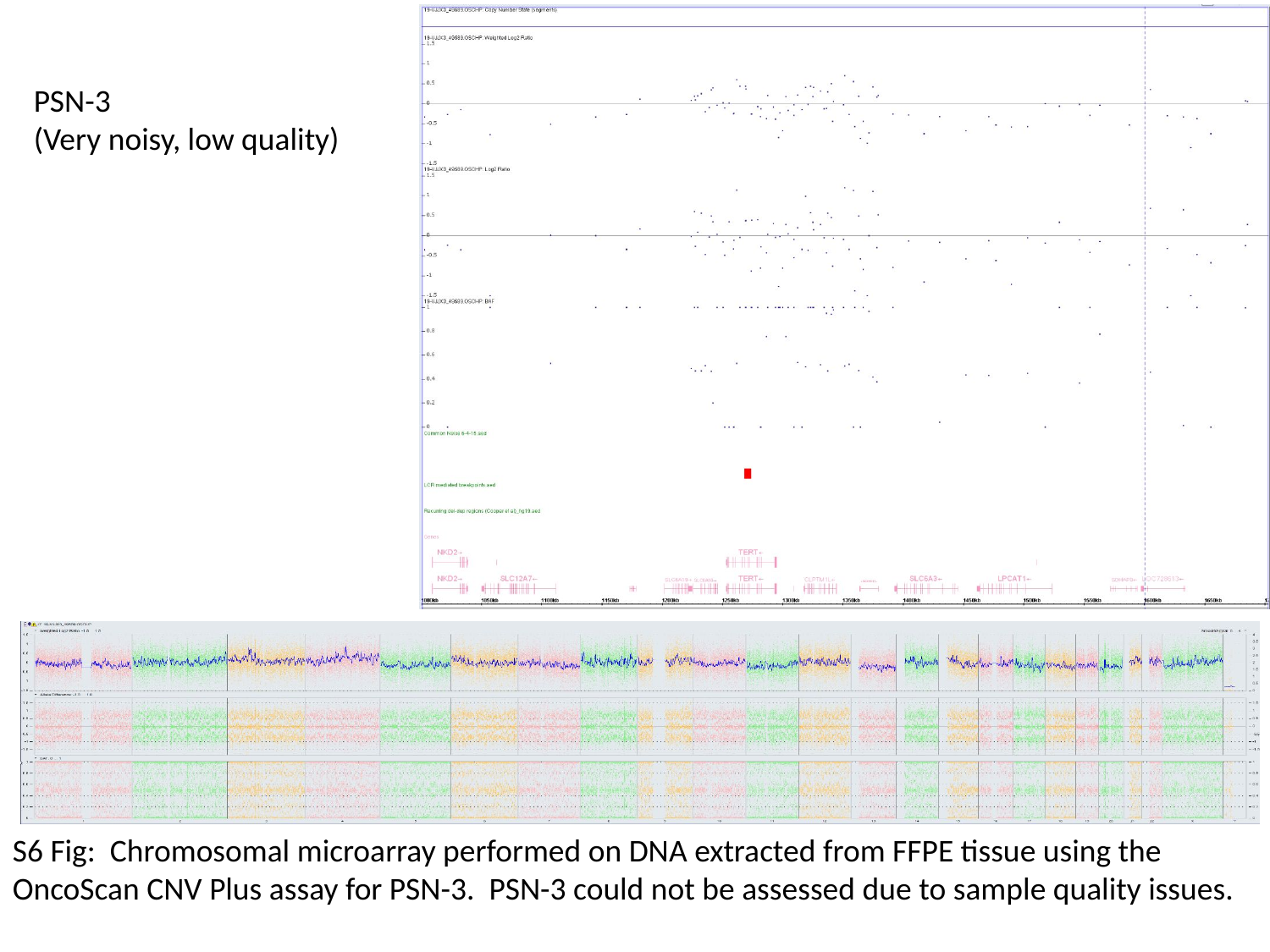

PSN-3
(Very noisy, low quality)
S6 Fig: Chromosomal microarray performed on DNA extracted from FFPE tissue using the OncoScan CNV Plus assay for PSN-3. PSN-3 could not be assessed due to sample quality issues.
