# Supplementary material for: LPCAT1-TERT fusions are uniquely recurrent in epithelioid trophoblastic tumors and positively regulate cell growth
Source: PLoS One. 2021 May 25;16(5):e0250518. doi: 10.1371/journal.pone.0250518 (PMC8148365; doi:10.1371/journal.pone.0250518)
Supplement: S8 Fig — PSTT-2 was classified as negative for copy number alterations and genomic deletions affecting LPCAT1 or TERT. (PPTX) [file pone.0250518.s008.pptx]

## Slide 1
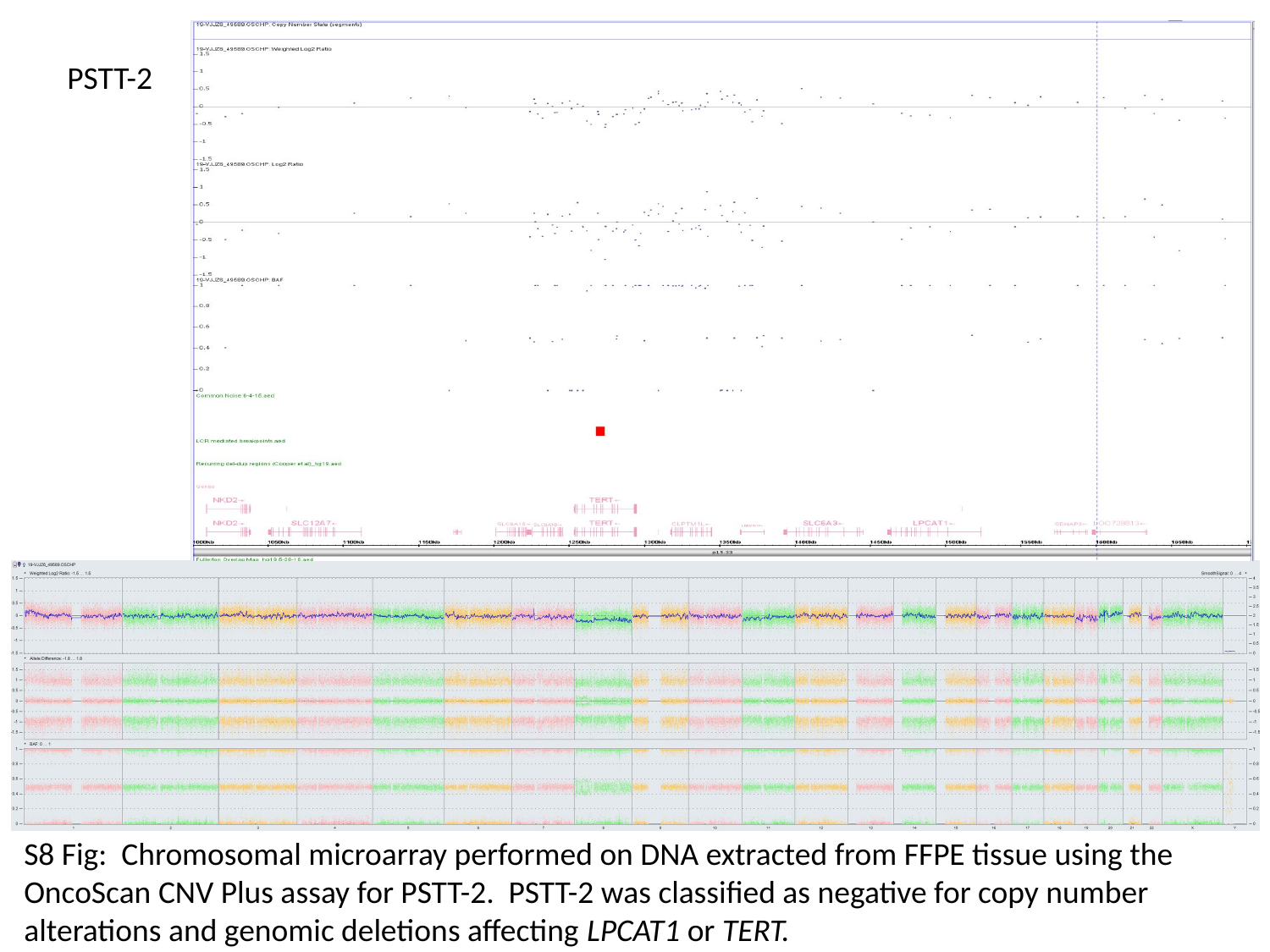

PSTT-2
S8 Fig: Chromosomal microarray performed on DNA extracted from FFPE tissue using the OncoScan CNV Plus assay for PSTT-2. PSTT-2 was classified as negative for copy number alterations and genomic deletions affecting LPCAT1 or TERT.
