# Supplementary material for: LPCAT1-TERT fusions are uniquely recurrent in epithelioid trophoblastic tumors and positively regulate cell growth
Source: PLoS One. 2021 May 25;16(5):e0250518. doi: 10.1371/journal.pone.0250518 (PMC8148365; doi:10.1371/journal.pone.0250518)
Supplement: S9 Fig — Ovarian and Uterine tissues are marked with a red asterisk (Median TPM of 0.0). (PPTX) [file pone.0250518.s009.pptx]

## Slide 1
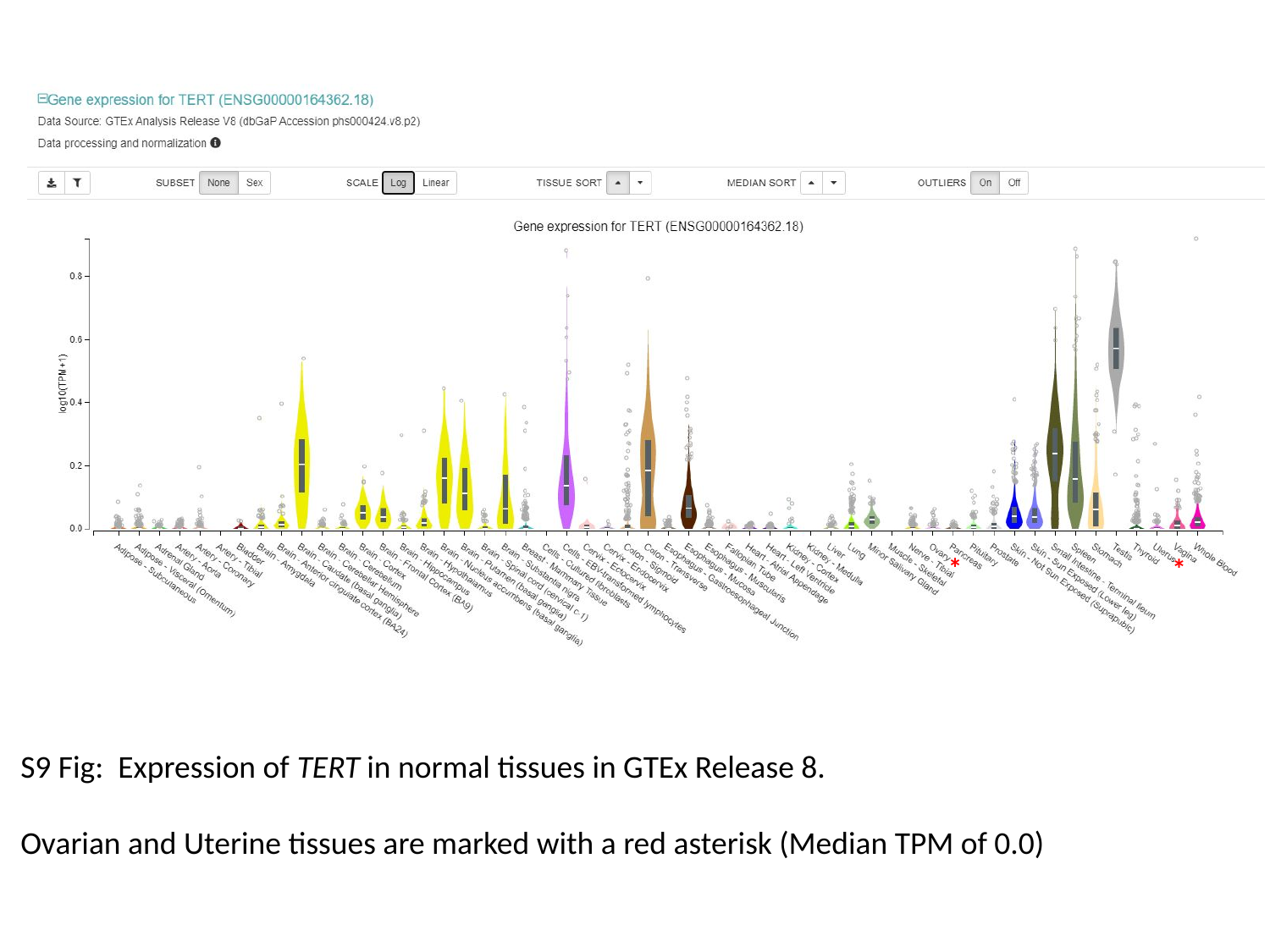

*
*
S9 Fig: Expression of TERT in normal tissues in GTEx Release 8.
Ovarian and Uterine tissues are marked with a red asterisk (Median TPM of 0.0)
