# Supplementary material for: LPCAT1-TERT fusions are uniquely recurrent in epithelioid trophoblastic tumors and positively regulate cell growth
Source: PLoS One. 2021 May 25;16(5):e0250518. doi: 10.1371/journal.pone.0250518 (PMC8148365; doi:10.1371/journal.pone.0250518)
Supplement: S10 Fig — TERT-LPCAT1 fusion is observed to localize mainly in the nucleus with some cytosolic localization (sample size = 2). (PPTX) [file pone.0250518.s010.pptx]

## Slide 1
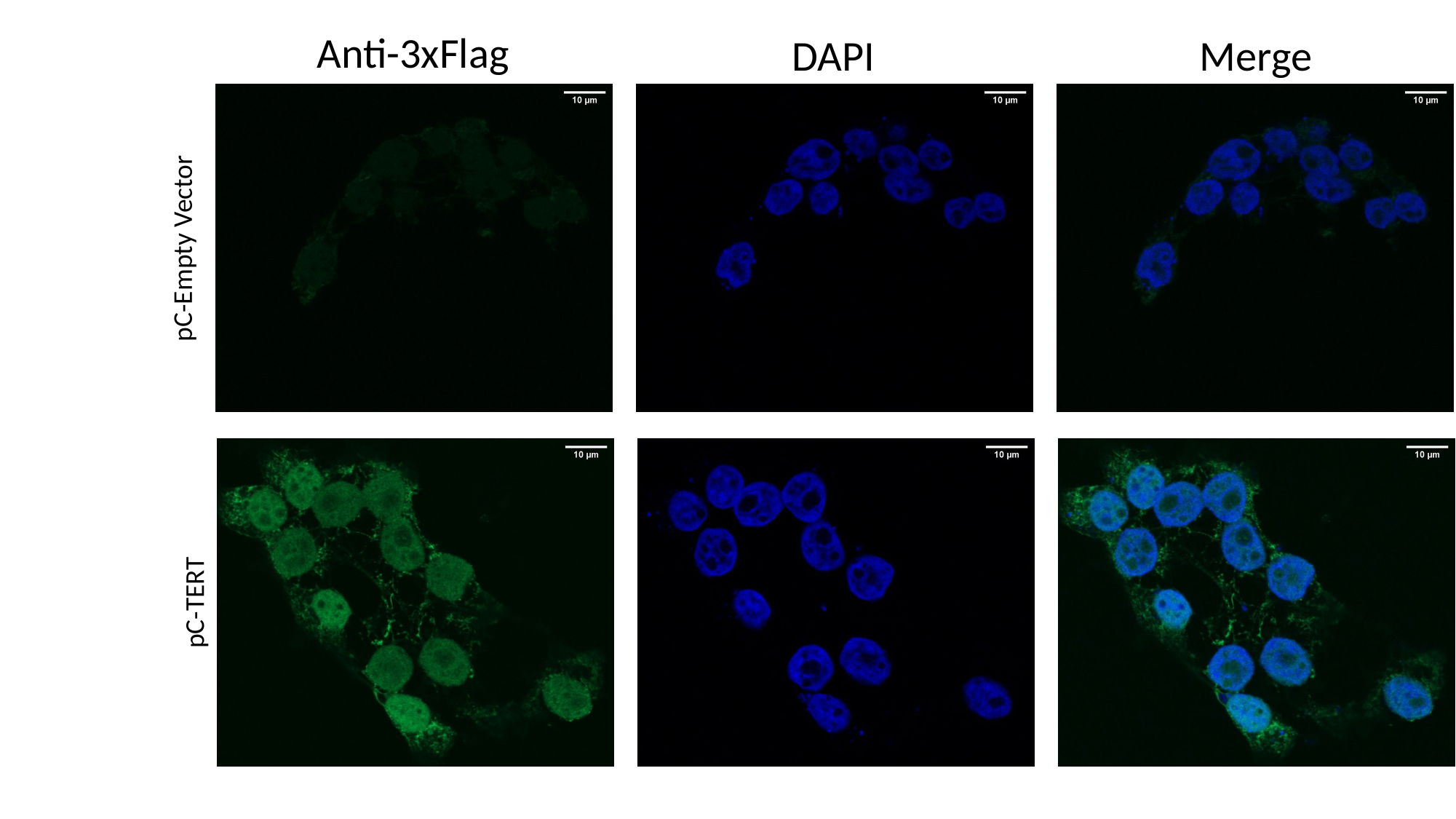

Anti-3xFlag
DAPI
Merge
pC-Empty Vector
pC-TERT

## Slide 2
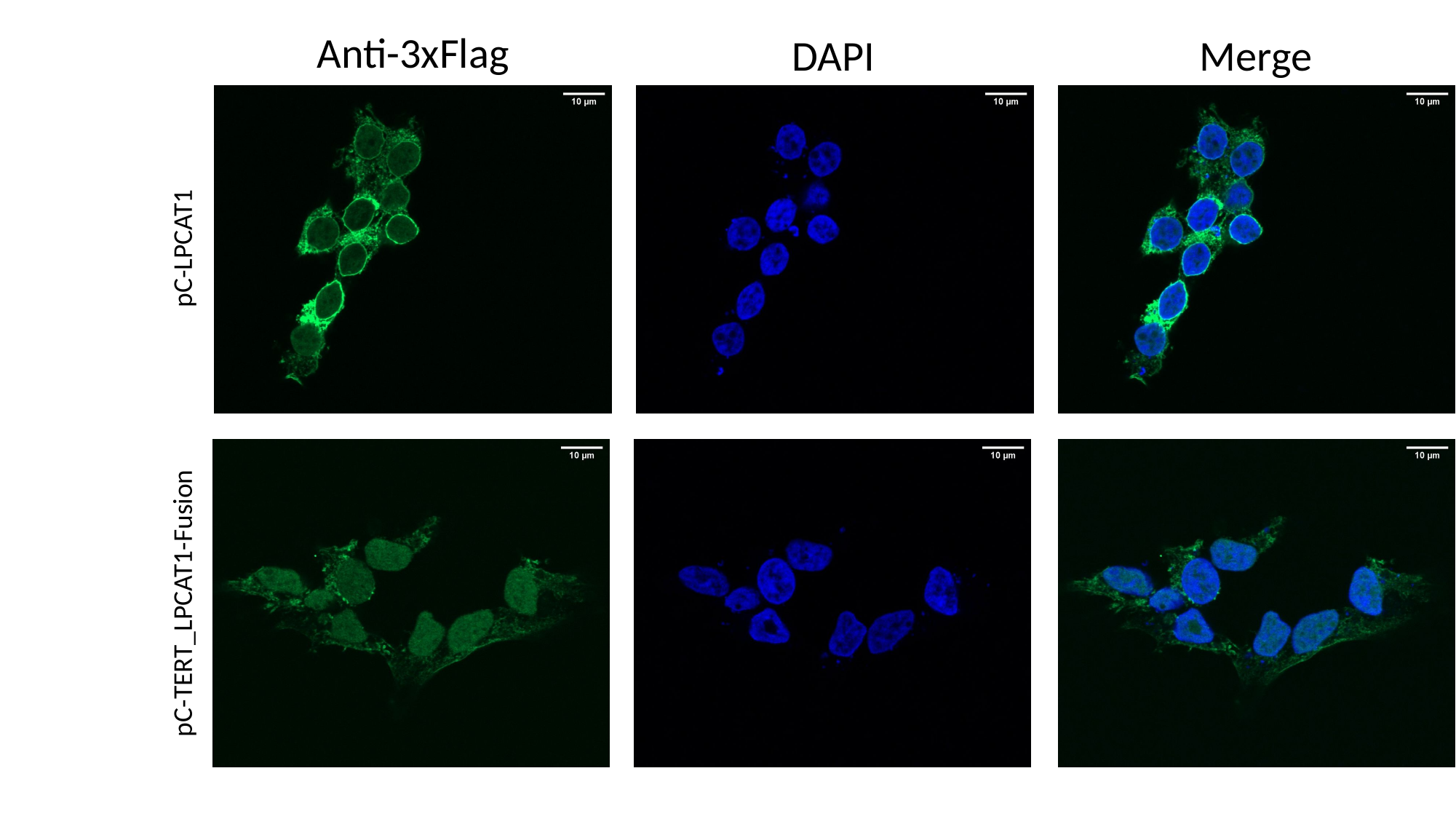

Anti-3xFlag
DAPI
Merge
pC-LPCAT1
pC-TERT_LPCAT1-Fusion
