# Supplementary material for: LPCAT1-TERT fusions are uniquely recurrent in epithelioid trophoblastic tumors and positively regulate cell growth
Source: PLoS One. 2021 May 25;16(5):e0250518. doi: 10.1371/journal.pone.0250518 (PMC8148365; doi:10.1371/journal.pone.0250518)

A

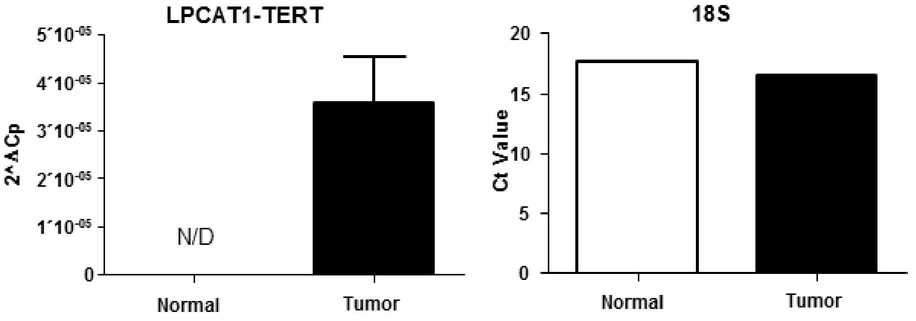

B

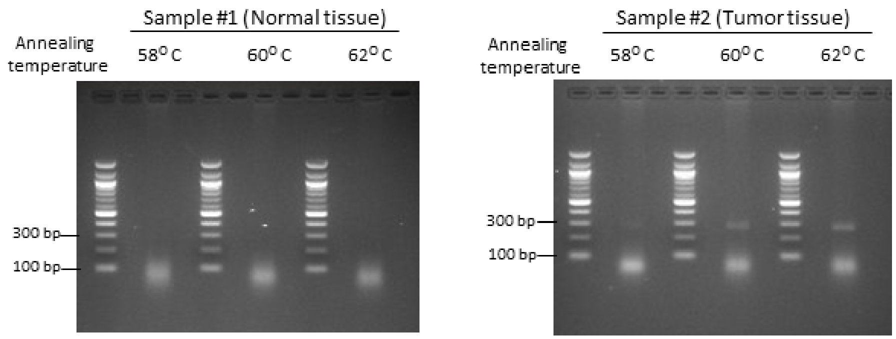

C

Sequencing

Sample #1 (62°C)
NNNNN

Sample #2 (62°C)
GGCTGACAGGACCTGTTCTTTTGAACGTGGTCTCCGTGACATAAAAGAAAGACCTGAGCAGCTCGACGACGTA
CACACTCATCAGCCAGTGCAGGAACTTGGCCAGGATCTCCTCACGCAGACGGTGCTCTGCGGCCGGAACACAG
CCAACCCTTCCCCAGATCGGGATGTCTCTGCTCTCTGCCTTCATCACGATGGAGGACATCGTCATGGTCACAGG
GATGGCGTCGAAGTAGGACGAGTGAGGCGA

B

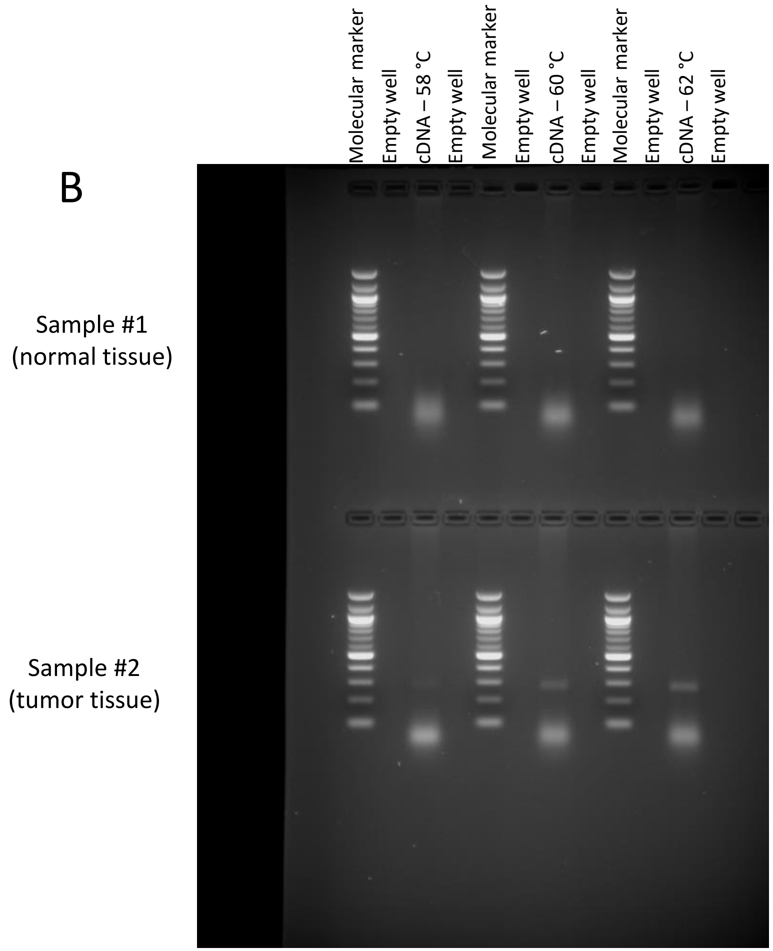

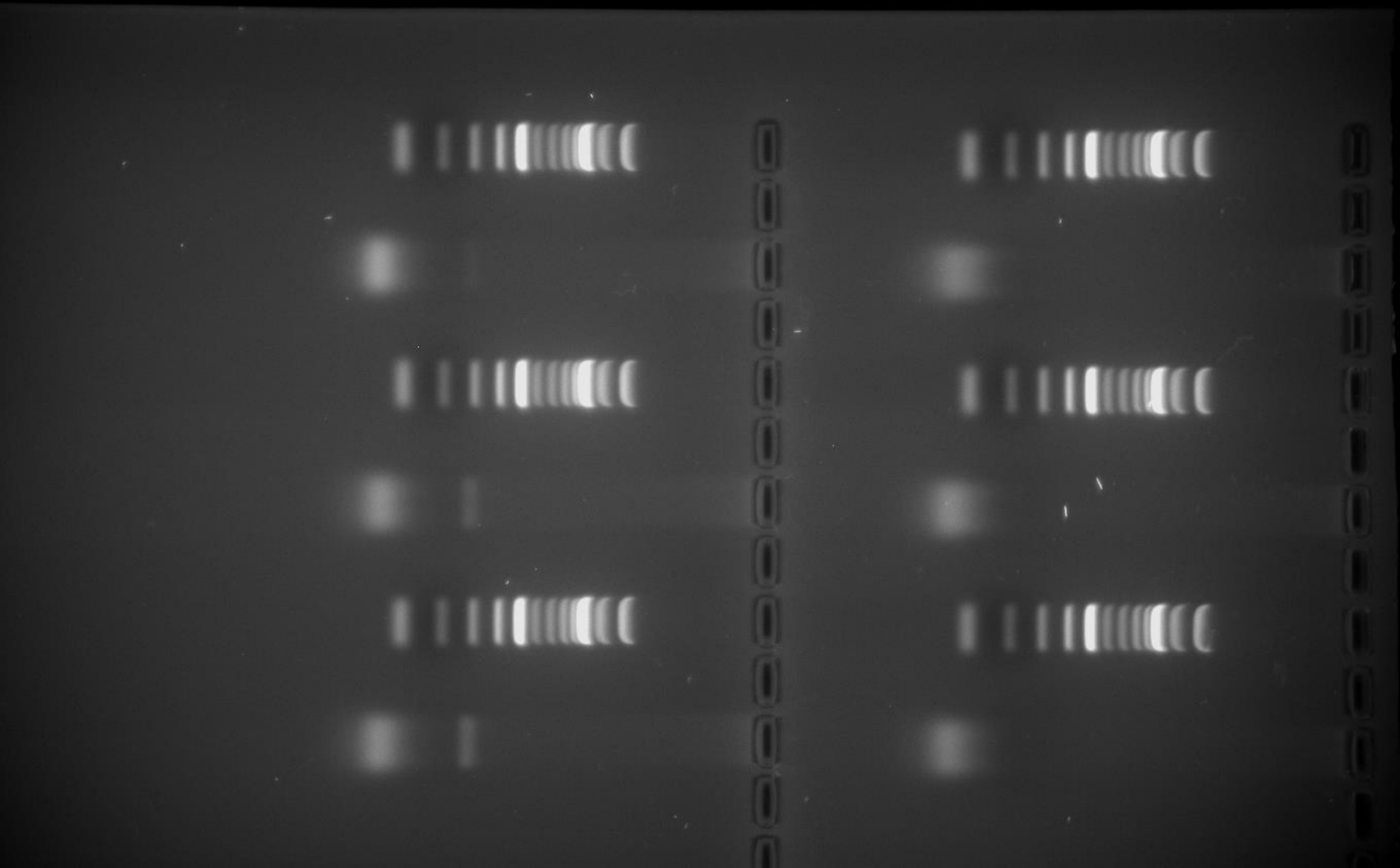

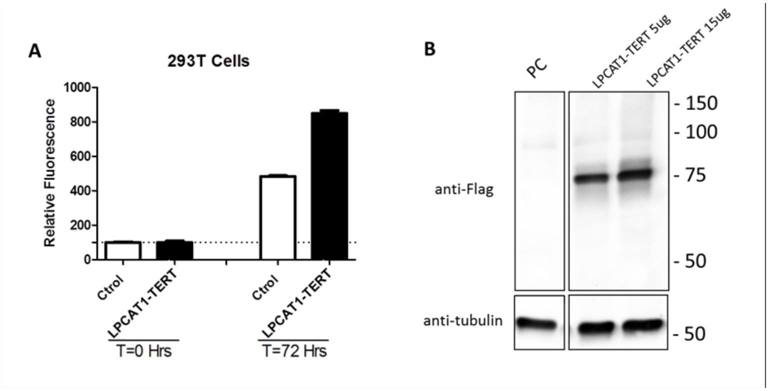

anti-Flag

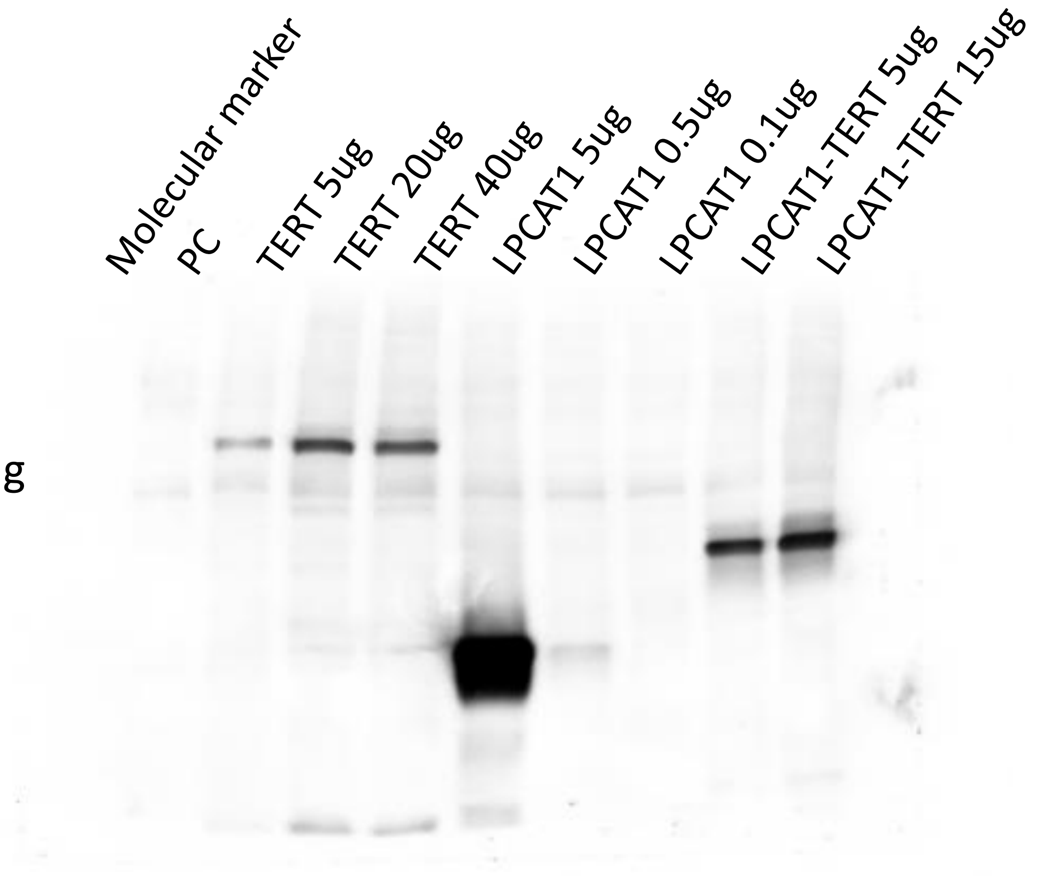

anti-tubulin

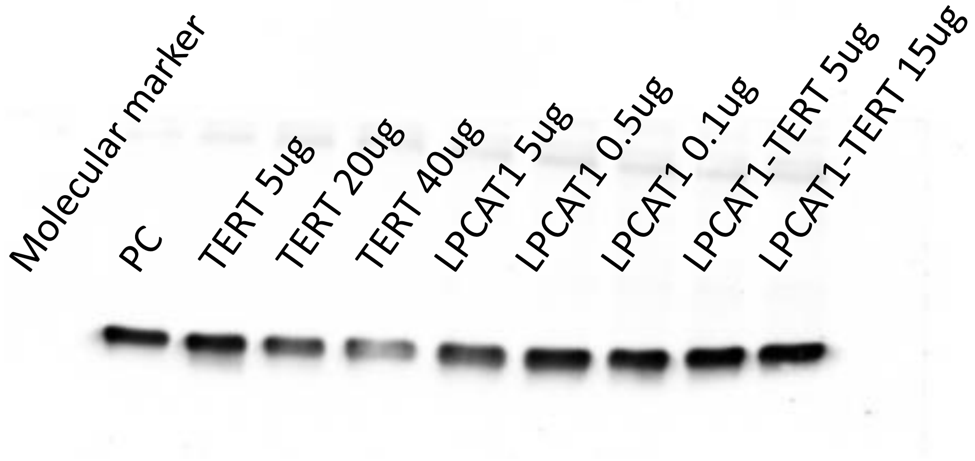

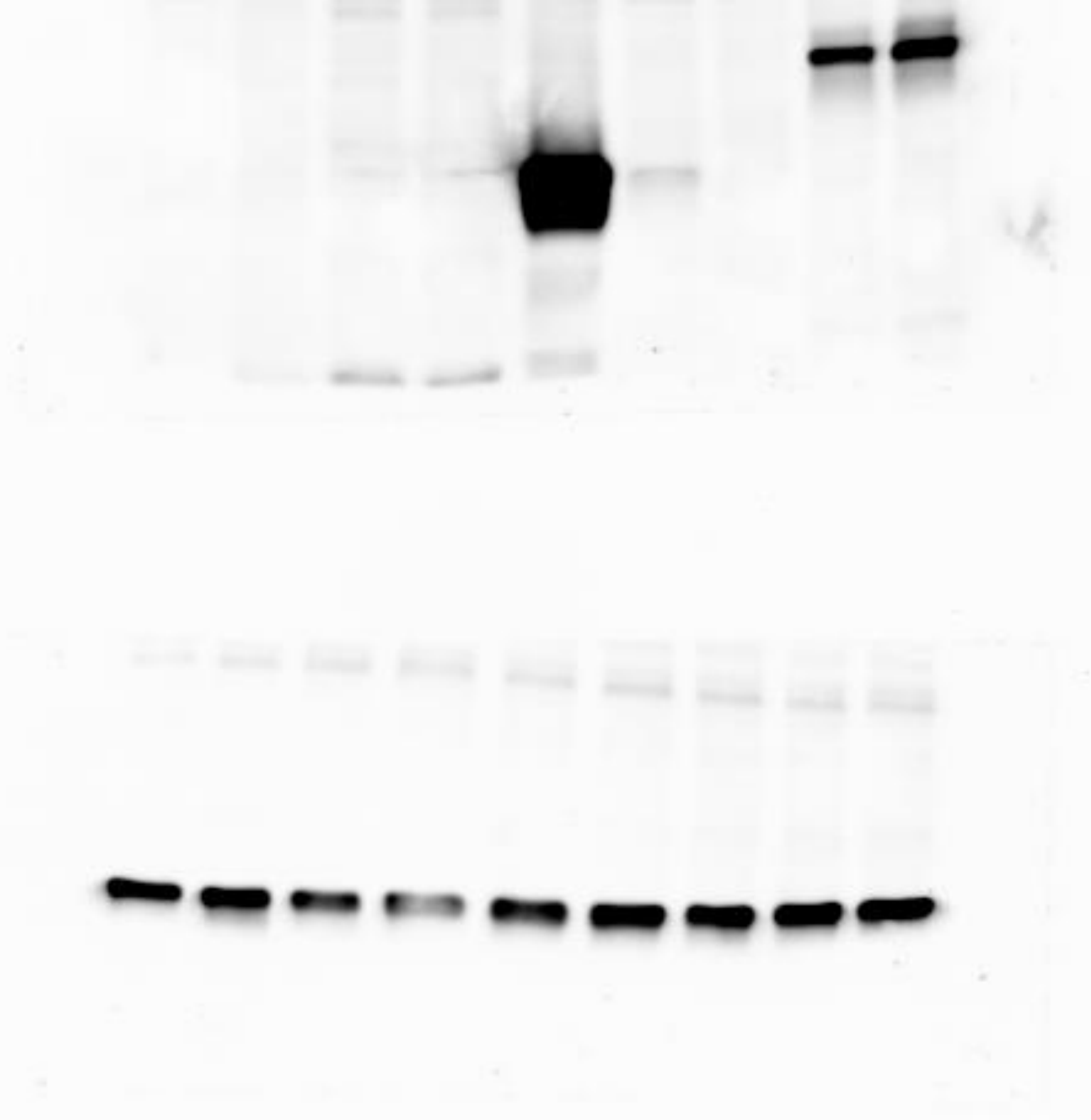

Supplement: S1 Raw images — (PDF) [file pone.0250518.s011.pdf]
