# Supplementary material for: LPCAT1-TERT fusions are uniquely recurrent in epithelioid trophoblastic tumors and positively regulate cell growth
Source: PLoS One. 2021 May 25;16(5):e0250518. doi: 10.1371/journal.pone.0250518 (PMC8148365; doi:10.1371/journal.pone.0250518)
Supplement: S1 Methods — (DOCX) [file pone.0250518.s012.docx]

**Plasmid Design**

**pC-LPCAT1_TERT-fus** was made by cloning the 538bp amplified LPCAT1 and 1.8kb TERT fusion sites into pSL1C that was linearized using HndIII. LPCAT1 was synthesized from GenScript (Clone ID: OHu09686) while TERT was purchased from AddGene (Plasmid #: 12245). The LPCAT1 and TERT fusion was amplified using primers GIB-LPCAT1-F1 [ACTACAAGGACGACGACGACAAGATGAGGCTGCGGGGATGC], GIB-LPCAT1-R1 [GCGGCCGGAACACAGCCAACCCTTCCCCAGATCGGGATGTCTCTG], GIB-TERT-F1 [CAGAGACATCCCGATCTGGGGAAGGGTTGGCTGTGTTCCGG], and GIB-pC-TERT-R1 [AGATGGCTGGCAACTAGAAGGCACAAAATCAGTCCAGGATGGTCTTGAAGTCTGA]. Using Gibson Assembly the LPCAT1_TERT fusion was cloned into the C-terminus of the 3XFlag tag and on the N-terminus of the BGH(A)_Ubiquitin promotor_tagGFP2.

**pC-LPCAT1-FL** plasmid was made by cloning a 1.6kb PCR amplification of LPCAT1 into pSL1C vector opened with HndIII. GIB-LPCAT1-F1 [ACTACAAGGACGACGACGACAAGATGAGGCTGCGGGGATGC] and GIB-pC-LPCAT1-R1 [AGATGGCTGGCAACTAGAAGGCACAAACTAATCCAGCTTCTTGCGAAC] primers were used to amplify the LPCAT1 fragment for Gibson Assembly into pSL1C.

**pC-TERT-FL** plasmid was made by cloning two PCR fragments of TERT (3.3kb) into pSL1C vector opened with HndIII. Due to the single nucleotide polymorphism (SNP) in the TERT at position 535 altering the amino acid from Aspartic Acid (D) > Glycine (G), primers were therefore used to correct the SNP. GIB-pA-TERT-F2 [ACTACAAGGACGACGACGACAAGATGCCGCGCGCTCCcc], GIB-TERT_SNP-R1 [CAGCCAAGCGCAGTCCCGCA], GIB-TERT_SNP-F1 [GAGCGTGCGGGACTGCGCTT], and GIB-pC-TERT-R1 [AGATGGCTGGCAACTAGAAGGCACAAAATCAGTCCAGGATGGTCTTGAAGTCTGA] primers were used to amplify/correct the SNP of TERT and then Gibson Assembled into the pSL1C vector.

**pSL1C** The plasmid was linearized with BspEI to allow for the amplified BGH(A) and ubiquitin C promoter. Primers used to amplify the BGH(A) were GIB-BGH(A)-F1 [CAAGGACGACGACGACAAGCTTTTGTGCCTTCTAGTTGCCAGCC] and MISC-GBH(A)-R1 [tcagctgcatcagcctgctattgtcttcccaatcc] from construct pKT2P-PTK (Clark, 2007). For the Ubiquitin C promoter primers GIB-UbProm-F1 [ACAATAGCAGGCTGATGCAGCTGAGATCTGGCCT] and GIB-UbProm-R1 [CTGATCCCCCACCAGAGCCTCCGTCCATGCTCGTCTAACAAAAAAGCCAAAAACGG] were used on plasmid pKUb-Tol2 (See below). With the overhang of the primers cloning was done using Gibson Assembly.

**pKTol2C-3XF_GFP** plasmid was made by cloning 3XFlag_GFP gene block with pKTol2C-tagGFP2 that was cut with EcoRI and NaeI. With the overhang of the primers for the 3XFlag_GFP gene block this allowed for the use of Gibson Assembly for cloning.

**3XFlag_GFP gene block** synthesized from IDT [GTGCTGTCTCATCATTTTGGCAAAGAATTCACTATGGACTACAAGGACCACGACGGCGACTACAAGGACCACGACATCGACTACAAGGACGACGACGACAAGCTTTCCGGAGGCTCTGGTGGGGGATCAGGAGGAGGCTCTGGTATGAGCGGAGGTGAGGAGCTGTTCGCCGGCATCGTGCCCGTGCTGATCGAGC]

**pKUb-Tol2** plasmid was created by cloning a 3.2kb vector backbone from pKUB-SB11 (Clark, 2007) with 1.9kb Tol2 PCR amplification from pCMV-Tol2 (Clark, 2007) plasmid. Tol2 was amplified using primers CDS-Tol2-F1 [TTGCTAGCCATGGAGGAAGTATGTGATTCATC] and CDC-Tol2-R1 [AAGGATCCTACTCAAAGTTGTAAAACCTCAGATT]. Then both the pKUB-SB11 and Tol2 PCR amplification were digested with BamHI and NheI and then ligated together.

**pKTol2C-tagGFP2** plasmid was made using pKTol2C-EGFP (Hoeppner et al., 2012) and cloned into 3.7kb N-terminus XhoI to C-terminus BglII site from pTagGFP2-N (Evrogen).
