# Supplementary material for: LPCAT1-TERT fusions are uniquely recurrent in epithelioid trophoblastic tumors and positively regulate cell growth
Source: PLoS One. 2021 May 25;16(5):e0250518. doi: 10.1371/journal.pone.0250518 (PMC8148365; doi:10.1371/journal.pone.0250518)
Supplement: Attachment — Submitted filename: PONE-D-20-36897.docx [file pone.0250518.s013.docx]

The work shows how TERT has a higher copy number in ETT lesions and also shows the presence of the LPCAT1-TERT fusion, which seems to appear in two of the three tumors of this type. The LPCAT-TERT fusion is not present in the other types of gestational trophoblastic diseases studied in this work.

The work is well structured, developed and discussed. This work opens the possibility of improving the diagnostic of EET, which is important.

I have a few comments about the work:

1. The fusion LPCAT1-TERT is a key part of the work. These genes and their main functions should be clarified in introduction. In fact, the paragraph written in discussion (lines 270 to line 275) could perfectly fit in the “Introduction section”.
2. Figure 1 would be much easier to understand if authors would use color arrows (or other symbols) to indicate the factors described in the figure legend 1 (i.e. nodules, expansile nests, etc.) Also, include a bar with the magnification of the image.
3. Legend of Figure 4. write what “n” indicates.
4. Line 284. “Furthermore, while assessment of telomere length in the presence or absence of LPCAT1-TERT has the potential to experimentally validate the absence of telomerase activity…”. In many tumors telomerase is activated but tumor cells have short telomeres, which are just minimally maintained so cells can continue to divide. The best evidence for telomerase activity is TRAP assay, which could be developed in a cell line which does not have telomerase activity, after LPCAT1-TERT transfection.
5. Since authors have the LPCAT-TERT-Flag construct, it would be interesting to know the localization cellular localization of the fusion protein, even if it were done in the 293T cells.
6. Authors find fusions between exons 1-3 of LPCAT1 and exons 1-2 of TERT gene. Is there any reason why these genomic regions are more prone to fusions than other regions?
7. Figure 2. The dotted line for ETT1 non-coding fusion is not easily distinguishable from the solid line. Perhaps authors could use another color to make it more visible.
8. Please, write “NA stands for not available” underneath Table 3. Alternatively, make it clear in the text (line 172): “not available (NA)”.
9. Just for clarity, in Figure 4 authors could write in the legend that Cp stands for “Crossing point” or whatever it may mean.
